# Nitrogen-containing bisphosphonates inhibit RANKL- and M-CSF-induced osteoclast formation through the inhibition of ERK1/2 and Akt activation

**DOI:** 10.1186/1423-0127-21-10

**Published:** 2014-02-03

**Authors:** Masanobu Tsubaki, Makiko Komai, Tatsuki Itoh, Motohiro Imano, Kotaro Sakamoto, Hirotaka Shimaoka, Tomoya Takeda, Naoki Ogawa, Kenji Mashimo, Daiichiro Fujiwara, Junji Mukai, Katsuhiko Sakaguchi, Takao Satou, Shozo Nishida

**Affiliations:** 1 Division of Pharmacotherapy, Kinki University School of Pharmacy, Kowakae, Higashi-Osaka 577-8502, Japan; 2 Department of Pathology, Kinki University School of Medicine, Osakasayama, Osaka, Japan; 3 Department of Surgery, Kinki University School of Medicine, Osakasayama, Osaka, Japan; 4 Department of Pharmacy, Izumi Municipal Hospital, Izumi, Osaka, Japan; 5 Department of Pharmacy, Japanese Red Cross Society Wakayama Medical Center, Wakayama, Japan

**Keywords:** Nitrogen-containing bisphosphonate, Geranylgeranyl pyrophosphate, MEK1/2, PI3K

## Abstract

**Background:**

Bisphosphonates are an important class of antiresorptive drugs used in the treatment of metabolic bone diseases. Recent studies have shown that nitrogen-containing bisphosphonates induced apoptosis in rabbit osteoclasts and prevented prenylated small GTPase. However, whether bisphosphonates inhibit osteoclast formation has not been determined. In the present study, we investigated the inhibitory effect of minodronate and alendronate on the osteoclast formation and clarified the mechanism involved in a mouse macrophage-like cell lines C7 and RAW264.7.

**Results:**

It was found that minodronate and alendronate inhibited the osteoclast formation of C7 cells induced by receptor activator of NF-κB ligand and macrophage colony stimulating factor, which are inhibited by the suppression of geranylgeranyl pyrophosphate (GGPP) biosynthesis. It was also found that minodronate and alendronate inhibited the osteoclast formation of RAW264.7 cells induced by receptor activator of NF-κB ligand. Furthermore, minodronate and alendornate decreased phosphorylated extracellular signal-regulated kinase 1/2 (ERK1/2) and Akt; similarly, U0126, a mitogen protein kinase kinase 1/2 (MEK1/2) inhibitor, and LY294002, a phosphatidylinositol 3-kinase (PI3K) inhibitor, inhibited osteoclast formation.

**Conclusions:**

This indicates that minodronate and alendronate inhibit GGPP biosynthesis in the mevalonate pathway and then signal transduction in the MEK/ERK and PI3K/Akt pathways, thereby inhibiting osteoclast formation. These results suggest a novel effect of bisphosphonates that could be effective in the treatment of bone metabolic diseases, such as osteoporosis.

## Background

Osteoclasts are multinucleated cells responsible for bone resorption. It is therefore critical to understand the regulatory mechanism of osteoclast formation and function to develop an effective treatment for metabolic bone diseases, such as osteoporosis [1]. Multinucleated osteoclasts are generated from hematopoietic precursor cells through the action of macrophage-colony stimulating factor (M-CSF) and receptor activator of NF-κB ligand (RANKL) [2-4]. These cytokines act on osteoclast precursor cells that express their receptors, c-fms and RANK, respectively. These receptors transmit osteoclastogenic signals through transcription-factor-activating intercellular kinase cascades, such as mitogen-activated protein kinases (MAPKs), phosphatidylinositol 3-kinase (PI3K)/Akt, and c-Src; these transcription factors include NF-κB, c-Fos/AP-1, and NFAT [5-7]. Consequently, it has been shown that mice deficient in NF-κB, c-Fos, NFAT, M-CSF, c-fms, RANK or RANKL cannot generate osteoclasts and develop osteopetrosis [8-11].

Nitrogen-containing bisphosphonates (N-BPs) are a class of drugs used in the treatment of osteoporosis and diseases associated with high bone turnover [12]. N-BPs have been shown to prevent formation of farnesyl pyrophosphate (FPP) and geranylgeranyl pyrophosphate (GGPP), through inhibition of FPP synthase and GGPP synthase, both enzymes in the mevalonate pathway [13-16]. A major effect of these agents is promotion of apoptosis of mature osteoclasts. Recently, N-BPs have been reported to inhibit osteoclast formation in vitro [17]. The mechanism of suppression of osteoclast formation has also been reported to involve the inhibition of GGPP biosynthesis. In addition, although there is a report that N-BPs also induce the inhibition of small GTPases prenylation [18], several characteristics of osteoclast precursor cells remain unclear. In the present study, we investigated the mechanism by which minodronate and alendronate inhibit osteoclast formation in the macrophage-like cell lines C7 and RAW264.7.

## Methods

### Materials

Minodronate was supplied from Astellas Pharmaceutical (Tokyo, Japan). Alendronate was purchased from LKT laboratories Inc. (St. Paul, MN, USA). These reagents were dissolved in phosphate-buffered saline (PBS; 0.05 M, pH7.4), filtrated through Syringe Filters (0.45 μm; IWAKI GLASS, Tokyo, Japan), and used for the various assays described below.

Farnesol (FOH) and geranylgeraniol (GGOH) were purchased from Sigma (St Louis, MO, USA). FOH and GGOH were dissolved in dry ethanol. U0126 and LY294002 were purchased from Promega (Southampton, Hants, UK) and dissolved in DMSO. The dissolved reagents were resuspended in PBS (0.05 M, pH 7.4) and filtered through syringe filters before use.

### Cell culture

We used C7 cells, which are mouse macrophage-like cells that have the ability to differentiate into osteoclasts [19,20]. The C7 cells were kindly provided by Dr. Shin-ichi Hayashi (Tottori University, Japan) and cultured in α-minimal essential medium (Sigma) supplemented with 10% fetal calf serum (Gibco, Carlsbad, CA), 50 ng/mL human recombinant M-CSF (Leukoprol; Kyowa Hakko, Shizuoka, Japan), 100 U/mL penicillin (Gibco) and 100 μg/mL streptomycin (Gibco), in an atmosphere containing 5% CO_2_. RAW264.7 cells were purchased from DS Pharma Biomedical (Osaka, Japan) and cultured in α-MEM supplemented with 10% FCS, 100 μg/mL penicillin, and 100 U/mL streptomycin in the presence of 5% CO_2_.

### Tartrate-resistant acid phosphatase (TRAP) staining

Cells were fixed with 10% formalin in PBS and were rinsed with HEPES-buffered solution (0.9% NaCl, 10 mM HEPES, pH 7.1). Cells were stained with Fast Red Violet LB (Sigma) dissolved in TRAP buffer (50 mM sodium acetate, 30 mM sodium tartrate, 0.1% Triton X-100, 100 μg naphthol AS-MX phosphate, pH 5.0) for 45 min at 37°C. Multinucleated osteoclasts were identified under light microscopy as TRAP-positive cells with three or more nuclei. The total number of TRAP-positive cells and the number of nuclei in each well were determined.

### Cell viability

Cell viability was assessed by the tetrazolium-dye method using a TetraColor ONE assay kit (WST-8 assay kit; Seikagaku, Tokyo, Japan). C7 cells (5 × 10^4^ cells/mL) were plated in 96-well plates and incubated with various concentrations of minodronate or alendronate for 12 days. Cultures were fed every three days by replacing with 50 μl of fresh medium with or without various concentrations of minodronate and alendronate. RAW264.7 cells (5 × 10^4^ cells/mL) were plated in 96-well plates and incubated with various concentrations of minodronate or alendronate for 7 days. Cultures were fed every three days by replacing with 50 μl of fresh medium with or without various concentrations of minodronate and alendronate. After both time points, absorbance was measured at 492 nm with a microplate reader (SK601, Seikagaku).

### Quantitative real-time polymerase chain reaction (PCR)

Total RNA was isolated using RNAiso (Takara Biomedical; Siga, Japan). One microgram of purified total RNA was used for the real-time PCR analysis with the PrimeScript RT reagent kit (Takara Biomedical). cDNA was subjected to quantitative real-time PCR by using SYBR Premix Ex *Taq* (Takara Biomedical) and the Thermal Cycler Dice Real Time system (Takara Biomedical) in a 96-well plate according to the manufacturer’s instructions. The PCR conditions for glyceraldehyde-3-phosphate dehydrogenase (GAPDH), calcitonin receptor (CTR), and cathepsin K were 94°C for 2 min; followed by 40 cycles of 94°C for 0.5 min, 50°C for 0.5 min, and 72°C for 0.5 min. The following primers were used: CTR, 5′-CCA TTC CTG TAC TTG GTT GGC-3′ (5′-primer) and 5′-AGC AAT CGA CAA GGA GTG AC-3′ (3′-primer); cathepsin K, 5′-GGA AGA AGA CTC ACC AGA AGC-3′ (5′-primer) and 5′-GTC ATA TAG CCG CCT CCA CAG-3′ (3′-primer); and GAPDH, 5′-ACT TTG TCA AGC TCA TTT-3′ (5′-primer) and 5′-TGC AGC GAA CTT TAT TG-3′ (3′-primer). As an internal control for each sample, the GAPDH gene was used for standardization. Cycle threshold (Ct) values were established, and the relative difference in expression from GAPDH expression was determined according to the 2^–∆∆Ct^ method of analysis and compared to the expression in control cells.

### Western blotting

C7 cells treated under various conditions were lysed with lysis buffer (20 mM Tris/HCl, pH 8.0, 150 mM NaCl, 2 mM EDTA, 100 mM NaF, 1% NP40, 1 μg/ml leupeptin, 1 μg/ml antipain and 1 mM PMSF). The protein content of this cell lysate was determined using the BCA protein assay kit (Pierce, Rockford, IL, USA). An aliquot of each extract (40 μg of protein) was fractionated by electrophoresis in an SDS-polyacrylamide gel and transferred to a polyvinylidene difluoride membranes (Amersham, Arlington Heights, IL, USA). Membranes were blocked with a solution containing 3% skim milk, and then incubated overnight at 4°C with each of the following antibodies: anti-phospho-extracellular signal-regulated kinase (ERK) 1/2 antibody, anti-phospho-Akt antibody, anti-phospho-p38MAPK antibody, anti-ERK1/2 antibody, anti-Akt antibody, and anti-p38MAPK antibody (Cell Signaling Technology, Beverly, MA, USA). Subsequently, the membranes were incubated for 1 h at room temperature with anti-rabbit IgG sheep antibody or anti-mouse IgG sheep antibody coupled to horseradish peroxidase (Amersham). Reactive proteins were visualized using a chemiluminescence (ECL-plus) kit (Amersham) according to the manufacturer’s instructions.

### Statistical analysis

All results are expressed as means and S.D. of several independent experiments. Multiple comparisons of the data were performed by ANOVA with Dunnett’s test. P values less than 5% were regarded as significant.

## Results

### Cytotoxicity against C7 and RAW264.7 cells

**Figure 1:**
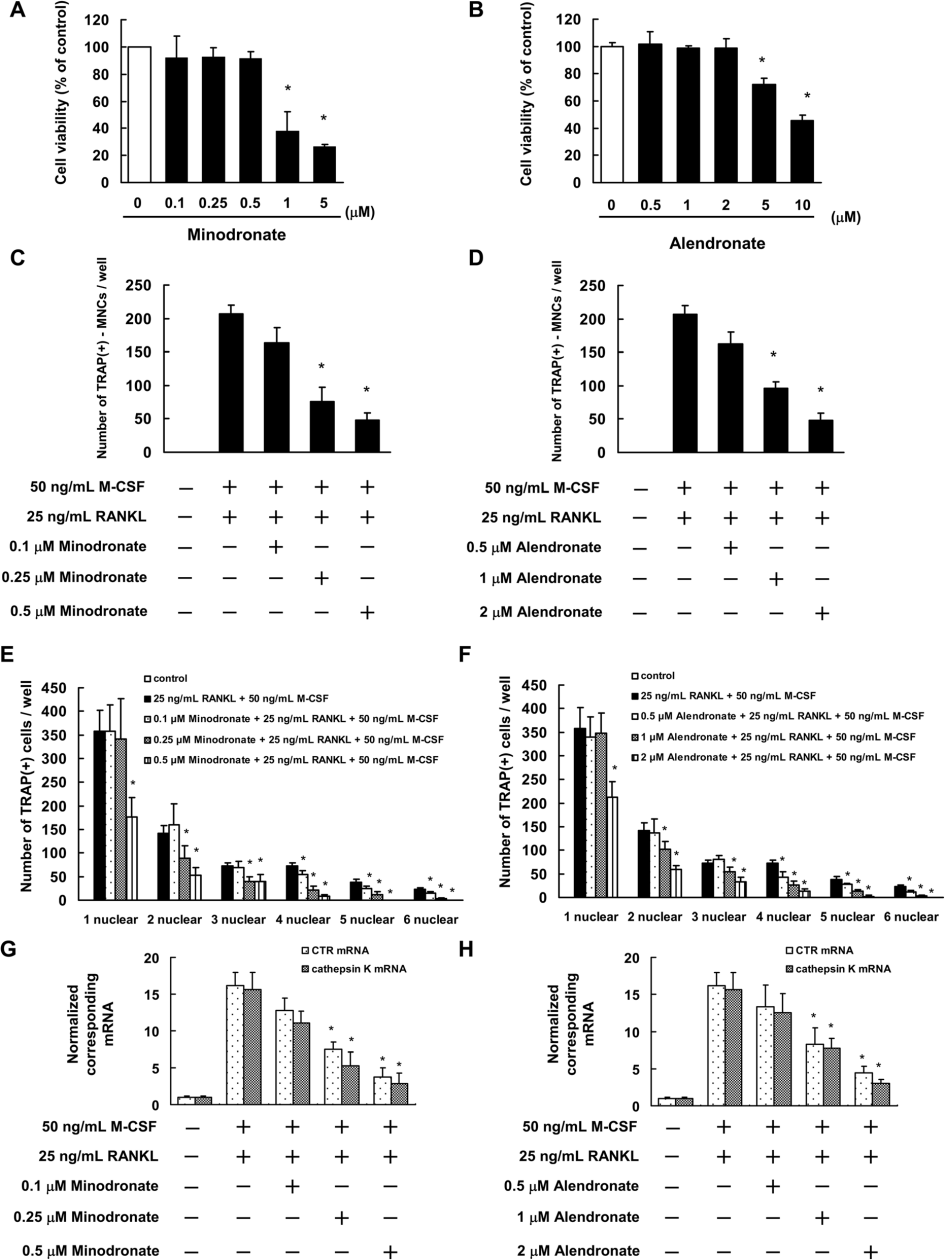
**Minodronate and alendronate inhibited osteoclast formation in C7 cells. (A, B)** Determination of the appropriate concentrations of minodronate **(A)** and alendronate **(B)** that are not cytotoxic to C7 cells. Cells (5000 cells/well) were incubated in 96-well plates for 24 h and then treated with various concentrations of minodronate and alendronate. After 12 days, cell viability was quantified by conducting WST-8 assays. The results are representative of 5 independent experiments. *P < 0.01 compared to the controls. **(C-F)** Inhibition of osteoclast formation by minodronate and alendronate. C7 cells were cultured for 12 days and then treated with 0.1, 0.25, or 0.5 μM minodronate **(C, E)** or 0.5, 1, or 2 μM alendronate **(D, F)**. Cells were cultured in the presence of 25 ng/mL RANKL plus 50 ng/mL M-CSF. Cultures were fed every 3 days by replacing with 500 μL of fresh medium with or without minodronate, alendronate, RANKL, and M-CSF. Cultures were fixed and stained for TRAP-positive multinucleated cells **(C, D)**, and TRAP-positive cells **(E, F)** per well was counted. These results are representative of 5 independent experiments. *P < 0.01 compared to 25 ng/mL RANKL plus 50 ng/mL M-CSF administration. **(G, H)** Inhibitory effect of minodronate and alendronate on RANKL and M-CSF-induced CTR and cathepsin K mRNA expression. C7 cells were treated with minodronate **(G)** or alendronate **(H)** with 25 ng/mL RANKL plus 50 ng/mL M-CSF for 12 days. Total RNA was extracted and the levels of CTR and cathepsin K mRNA expression were determined by real-time PCR. The results are expressed as the ratio of treated to control samples after normalization to GAPDH mRNA levels. The results are representative of 4 independent experiments. *P < 0.01 compared to 25 ng/mL RANKL plus 50 ng/mL M-CSF administration.

The cytotoxic effects of minodronate and alendronate on C7 cells were measured by WST-8 assay. The results showed that minodronate did not affect cell viability at a concentration of 0.1 μM to 0.5 μM for 12 days (Figure 1A). We also found that alendronate did not affected cell viability at a concentration of 0.5 μM to 2 μM for 12 days (Figure 1B). On the basis of these results, 0.1 to 0.5 μM were determined to be non-cytotoxic concentrations of minodronate, and 0.5 to 2 μM were determined to be non-cytotoxic concentrations of alendronate.

**Figure 2:**
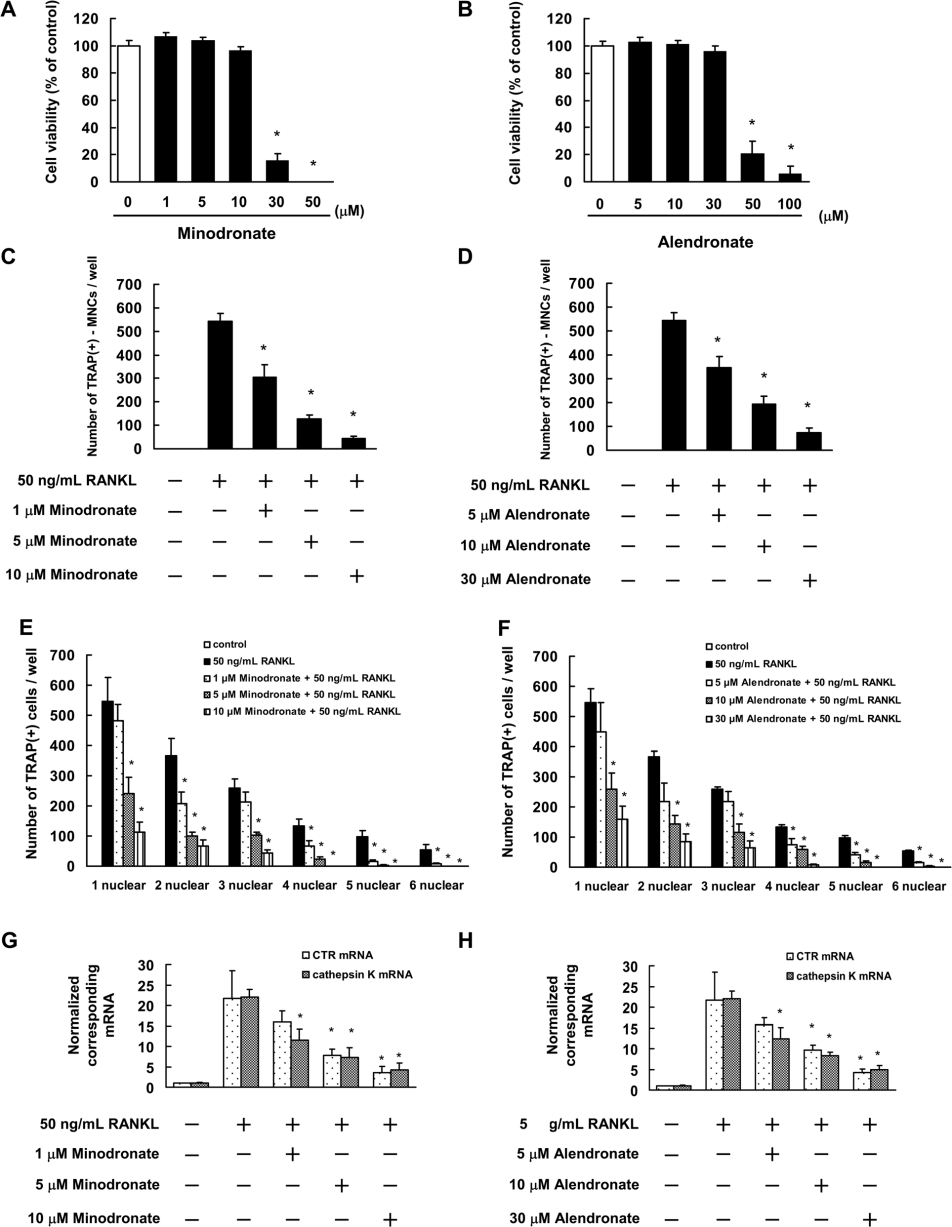
**Minodronate and alendronate inhibited osteoclast formation in RAW264.7 cells. (A, B)** Determination of the appropriate concentrations of minodronate **(A)** and alendronate **(B)** that are not cytotoxic to RAW264.7 cells. Cells (5000 cells/well) were incubated in 96-well plates for 24 h and then treated with various concentrations of minodronate and alendronate. After 7 days, cell viability was quantified by conducting WST-8 assays. The results are representative of 4 independent experiments. *P < 0.01 compared to the controls. **(C-F)** Inhibition of osteoclast formation by minodronate and alendronate. RAW264.7 cells were cultured for 12 days and then treated with 1, 5, or 10 μM minodronate **(C, E)** or 5, 10, or 20 μM alendronate **(D, F)**. Cells were cultured in the presence of 50 ng/mL RANKL. Cultures were fed every 2 days by replacing with 500 μL of fresh medium, with or without minodronate, alendronate, and RANKL. Cultures were fixed and stained for TRAP-positive multinucleated cells **(C, D)**, and TRAP-positive cells **(E, F)** per well was counted. These results are representative of 4 independent experiments. *P < 0.01 compared to 50 ng/mL RANKL administration. **(G, H)** Inhibitory effect of minodronate and alendronate on RANKL and M-CSF-induced CTR and cathepsin K mRNA expression. RAW264.7 cells were treated with minodronate **(G)** and alendronate **(H)** with 50 ng/mL RANKL for 7 days. Total RNA was extracted, and the CTR and cathepsin K mRNA levels were determined by real-time PCR. The results are expressed as the ratio of treated to control samples after normalization to GAPDH mRNA levels. The results are representative of 4 independent experiments. *P < 0.01 compared to 50 ng/mL RANKL administration.

Next, we examine the cytotoxic effects of minodronate and alendronate on RAW264.7 cells. The results showed that minodronate did not affect cell viability at a concentration of 1 μM to 10 μM for 7 days (Figure 2A). We also found that alendronate did not affected cell viability at a concentration of 5 μM to 30 μM for 7 days (Figure 2B). On the basis of these results, 1 to 10 μM were determined to be non-cytotoxic concentrations of minodronate, and 5 to 30 μM were determined to be non-cytotoxic concentrations of alendronate.

### Minodronate and alendronate inhibited osteoclast formation through suppression of GGPP biosynthesis

We investigated the effect of minodronate and alendronate on osteoclast formation in the presence of 25 ng/mL RANKL plus 50 ng/mL M-CSF, with or without minodronate and alendronate in C7 cells. C7 cells were seeded in 24-well plates (5000 cells/mL, 1 mL/well) in the presence of 25 ng/mL RANKL plus 50 ng/mL M-CSF with or without minodronate and alendronate. The culture medium was replaced every 3 days. Generation of TRAP-positive multinucleated cells increased in C7 cells in the presence of 25 ng/mL RANKL plus 50 ng/mL M-CSF. On the other hand, minodronate and alendronate inhibited the generation of TRAP-positive multinucleated cells in a concentration-dependent manner (Figure 1C, D). We also investigated the effect of minodronate and alendronate on the osteoclastogenesis process. Minodronate and alendronate inhibited the cell fusion process in a concentration-dependent manner (Figure 1E, F). In addition, minodronate and alendronate inhibited the RANKL- and M-CSF-induced mRNA expression of the osteoclast markers CTR and cathepsin K (Figure 1G, H).

Next, we investigated whether minodronate and alendronate suppress osteoclast formation in RAW264.7 cells. RAW264.7 cells were seeded in 24-well plates (10,000 cells/mL, 1 mL/well) in the presence of 50 ng/mL RANKL, with or without minodronate and alendronate. The culture medium was replaced every 2 days. The results showed that minodronate and alendronate inhibited the RANKL-induced TRAP-positive multinucleated cell formation and cell fusion process in a concentration-dependent manner (Figure 2C-F). In addition, minodronate and alendronate inhibited the RANKL-induced mRNA expression of CTR and cathepsin K (Figure 2G, H). These results indicated that N-BPs suppressed osteoclast formation through inhibition of cell fusion in osteoclast precursor cells.

**Figure 3:**
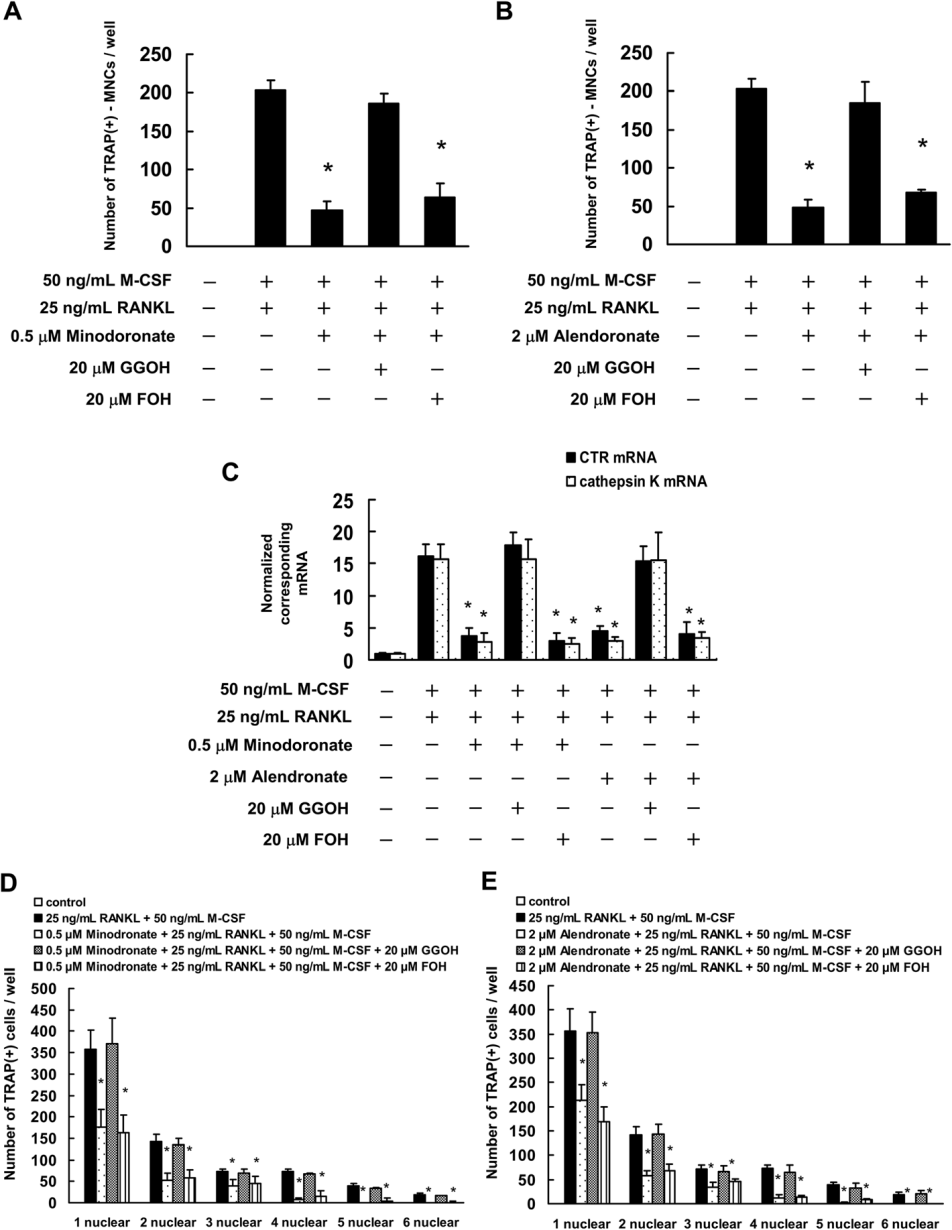
**Minodronate and alendronate inhibited osteoclast formation via suppression of GGPP biosynthesis in C7 cells.** C7 cells were pretreated with 20 μM FOH or 20 μM GGOH for 4 h and were then treated with 0.5 μM minodronate or 2 μM alendronate and 25 ng/mL RANKL plus 50 ng/mL M-CSF for 12 days. Cultures were fed every 3 days by replacing with 500 μL of fresh medium, with or without minodronate, alendronate, RANKL, M-CSF, FOH, and GGOH. **(A, B)** Inhibitory effect of mevalonate pathway intermediates FOH or GGOH on the inhibition of osteoclast formation by minodronate **(A)** and alendronate **(B)**. Cultures were fixed and stained for TRAP-positive multinucleated cells, and the number of cells per well was counted. These results are representative of 5 independent experiments. *P < 0.01 compared to 25 ng/mL RANKL plus 50 ng/mL M-CSF administration. **(C)** CTR and cathepisin K mRNA expression in C7 cells that were treated with minodronate or alendronate along with FOH or GGOH. Total RNA was extracted, and the CTR and cathepsin K mRNA levels were determined by real-time PCR. The results are expressed as the ratio of treated to control samples after normalization to GAPDH mRNA levels. The results are representative of 4 independent experiments. *P < 0.01 compared to 25 ng/mL RANKL plus 50 ng/mL M-CSF administration. **(D, E)** Cultures were fixed and stained for TRAP-positive cells, and the number of cells per well was counted. These results are representative of 4 independent experiments. *P < 0.01 compared to 25 ng/mL RANKL plus 50 ng/mL M-CSF administration.

**Figure 4:**
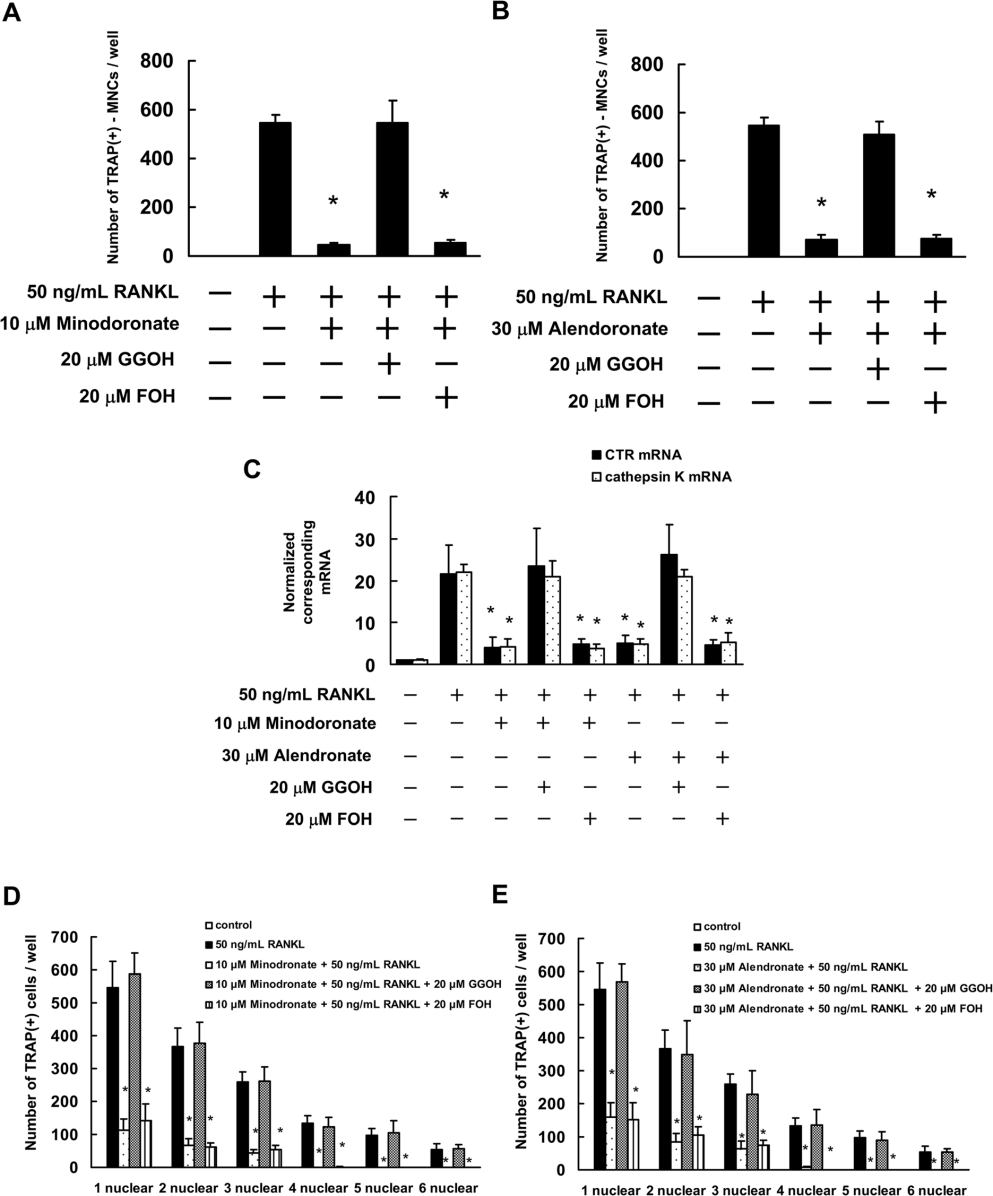
**Minodronate and alendronate inhibited osteoclast formation via suppression of GGPP biosynthesis in RAW264.7 cells.** RAW264.7 cells were pretreated with 20 μM FOH or 20 μM GGOH for 4 h and then treated with 10 μM minodronate or 30 μM alendronate, and 50 ng/mL RANKL for 7 days. Cultures were fed every 2 days by replacing with 500 μL of fresh medium, with or without minodronate, alendronate, RANKL, FOH, and GGOH. **(A, B)** Inhibitory effect of mevalonate pathway intermediates FOH or GGOH on the inhibition of osteoclast formation by minodronate **(A)** or alendronate **(B)**. Cultures were fixed and stained for TRAP-positive multinucleated cells, and the number of cells per well was counted. These results are representative of 5 independent experiments. *P < 0.01 compared to 50 ng/mL RANKL administration. **(C)** CTR and cathepsin K mRNA expression in RAW264.7 cells that were treated with minodronate or alendronate along with FOH or GGOH. Total RNA was extracted, and the CTR and cathepsin K mRNA levels were determined by real-time PCR. The results are expressed as the ratio of treated to control samples after normalization to GAPDH mRNA levels. The results are representative of 4 independent experiments. *P < 0.01 compared to 50 ng/mL RANKL administration. **(D, E)** Cultures were fixed and stained for TRAP-positive cells, and the number of cells per well was counted. These results are representative of 4 independent experiments. *P < 0.01 compared to 50 ng/mL RANKL administration.

Previous studies have demonstrated that minodronate and alendronate are capable of interfering with the mevalonate pathway in many cell types, including osteoclastic cells. Minodronate inhibits the synthesis of FPP or GGPP, two mevalonate pathway intermediates, and as a consequence, decreases prenylation of small GTPases such as Ras and Rho [21-25]. Therefore, we next investigated the possibility that minodronate and alendronate inhibit osteoclast formation through suppression of FPP or GGPP biosynthesis in C7 and RAW264.7 cells. Minodronate and alendronate inhibited osteoclast formation, whereas in combination with GGOH, generation of TRAP-positive multinucleated cells, mRNA expression of CTR and cathepsin K, and osteoclast nuclei numbers were restored to the levels observed in the presence of RANKL and M-CSF in C7 cells (Figure 3). By contrast, co-treatment with FOH did not significantly reverse the effects of minodronate and alendronate on osteoclast formation. In addition, similar to osteoclast formation in RAW264.7 cells, following the combination of GGOH with minodronate or alendronate, generation of TRAP-positive multinucleated cells, mRNA expression of CTR and cathepsin K, and osteoclast nuclei numbers were restored to the levels observed in the presence of RANKL (Figure 4). This suggests that the inhibition of osteoclast formation in C7 and RAW264.7 cells receiving N-BPs was due to inhibition of GGPP biosynthesis.

### Minodronate and alendronate inhibited RANKL- and M-CSF-induced ERK1/2 and Akt activation in C7 cells

**Figure 5:**
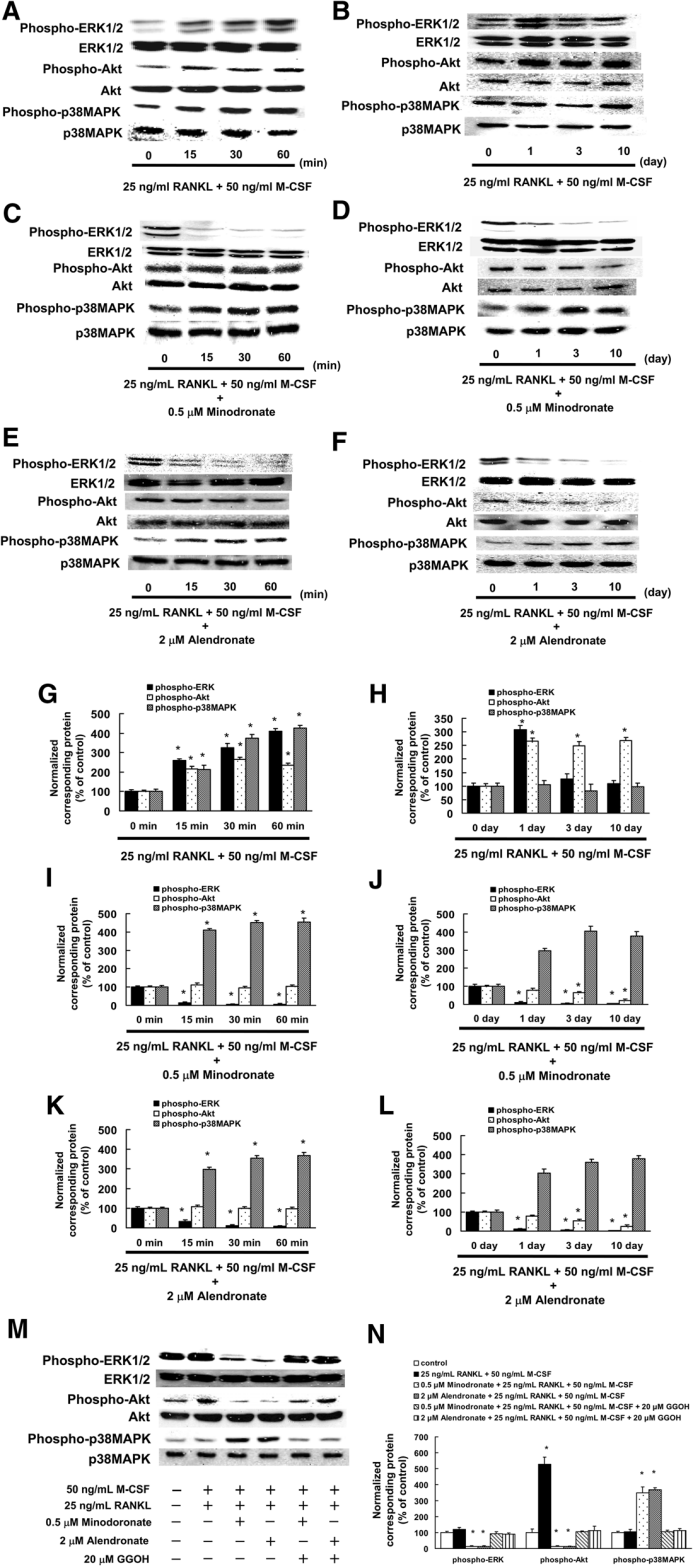
**RANKL plus M-CSF induced activation of ERK1/2 and Akt in C7 cells, which could be inhibited by minodronate and alendronate. (A, B)** C7 cells were cultured in the presence of 25 ng/mL RANKL plus 50 ng/mL M-CSF for 15, 30, and 60 min **(A)** or 1, 3, and 10 days **(B)**. **(C, D)** C7 cells were treated with 0.5 μM minodronate for 24 h. Cells were cultured in the presence of 25 ng/mL RANKL plus 50 ng/mL M-CSF for 15, 30, and 60 min **(C)** or 1, 3, and 10 days **(D)**. **(E, F)** C7 cells were treated with 2 μM alendronate for 24 h. Cells were cultured in the presence of 25 ng/mL RANKL plus 50 ng/mL M-CSF for 15, 30, and 60 min **(E)** or 1, 3, and 10 days **(F)**. Whole cell lysates were generated and immunoblotted with an antibody against phosphorylated ERK1/2 (phospho-ERK1/2), phosphorylated Akt (phospho-Akt), phosphorylated p38MAPK (phospho-p38MAPK), ERK1/2, Akt, and p38MAPK. **(G–L)** Quantification of the amount of phospho-ERK1/2, phospho-Akt, or phospho-p38MAPK normalized to the amount of total ERK1/2, Akt, or p38MAPK, respectively. The results are representative of 5 independent experiments. *P < 0.01 compared to controls. **(M)** ERK1/2, Akt, and p38MAPK activation in C7 cells, to which minodronate and alendronate were administered with or without the addition of GGOH. Phospho-ERK1/2, phospho-Akt, phospho-p38MAPK, ERK1/2, Akt, and p38MAPK levels were determined by immunoblotting analysis of the whole cell lysate. **(N)** Quantification of the amount of phospho-ERK1/2, phospho-Akt, or phospho-p38MAPK normalized to the amount of total ERK1/2, Akt, or p38MAPK, respectively. The results are representative of 4 independent experiments. *P < 0.01 compared to controls.

To investigate the molecular mechanisms of minodronate and alendronate in C7 cells, we examined phosphorylated ERK1/2, Akt and p38MAPK by Western blot analysis. An increase in phosphorylation of ERK1/2, Akt, and p38MAPK were observed 15–60 min after RANKL plus M-CSF treatments (Figure 5A, G). We also observed that an increase in phosphorylation of ERK1/2 was observed 1 day after RANKL plus M-CSF treatments. In the phosphorylation of Akt, a consecutive increase was observed 1–10 days after RANKL plus M-CSF treatment, compared to the vehicle (PBS-treated). A substantial change in phosphorylated p38MAPK was not observed after RANKL plus M-CSF treatments (Figure 5B, H).

The administration of minodronate and alendronate did not lead to any increase in the phosphorylation of ERK1/2 after RANKL plus M-CSF treatments. On the contrary, this protein showed a decrease in phosphorylation 15–60 min after RANKL plus M-CSF treatments. After administration of minodronate, there was no consecutive increase in the phosphorylation of Akt. However, minodronate and alendronate did not inhibited the level of p38MAPK phosphorylation (Figure 5C, E, I, K). Moreover, we found that the administration of minodronate and alendronate decreased in phosphorylation of ERK1/2 at 1–10 days after RANKL plus M-CSF treatments. After administration of minodronate, there was no consecutive increase in the phosphorylation of Akt, and a decrease from the vehicle level was confirmed at 10 days after RANKL plus M-CSF treatments. A change in phosphorylated p38MAPK increased by minodronate administration after RANKL plus M-CSF treatments (Figure 5D, F, J, L).

We also investigated whether administration of minodronate or alendronate alone caused a change in phosphorylated ERK1/2, Akt, and p38MAPK. After administration of minodronate or alendronate alone, phosphorylated ERK1/2 and Akt showed a sufficient decrease compared with that of the vehicle (PBS-treated) from day 1 through day 10. A change in phosphorylated p38MAPK increased by minodronate or alendronate administration (data not shown).

We then administered minodronate or alendronate in combination with GGOH to investigate whether the inhibition of ERK1/2 and Akt activation in C7 cells was due to the inhibitory action of minodronate and alendronate on GGPP biosynthesis via their mechanism of action. Minodronate and alendronate inhibited the activation of ERK1/2 and Akt, whereas in combination with GGOH, the activation levels of these signal transduction molecules were restored to the degree observed in RANKL and M-CSF-treated cells (Figure 5M, N). These observations suggest that the inhibition of ERK1/2 and Akt activation in C7 cells treated with minodronate and alendronate was due to the inhibition of GGPP biosynthesis.

### Effects of U0126 and LY294002 administration on osteoclast formation of C7 cells

**Figure 6:**
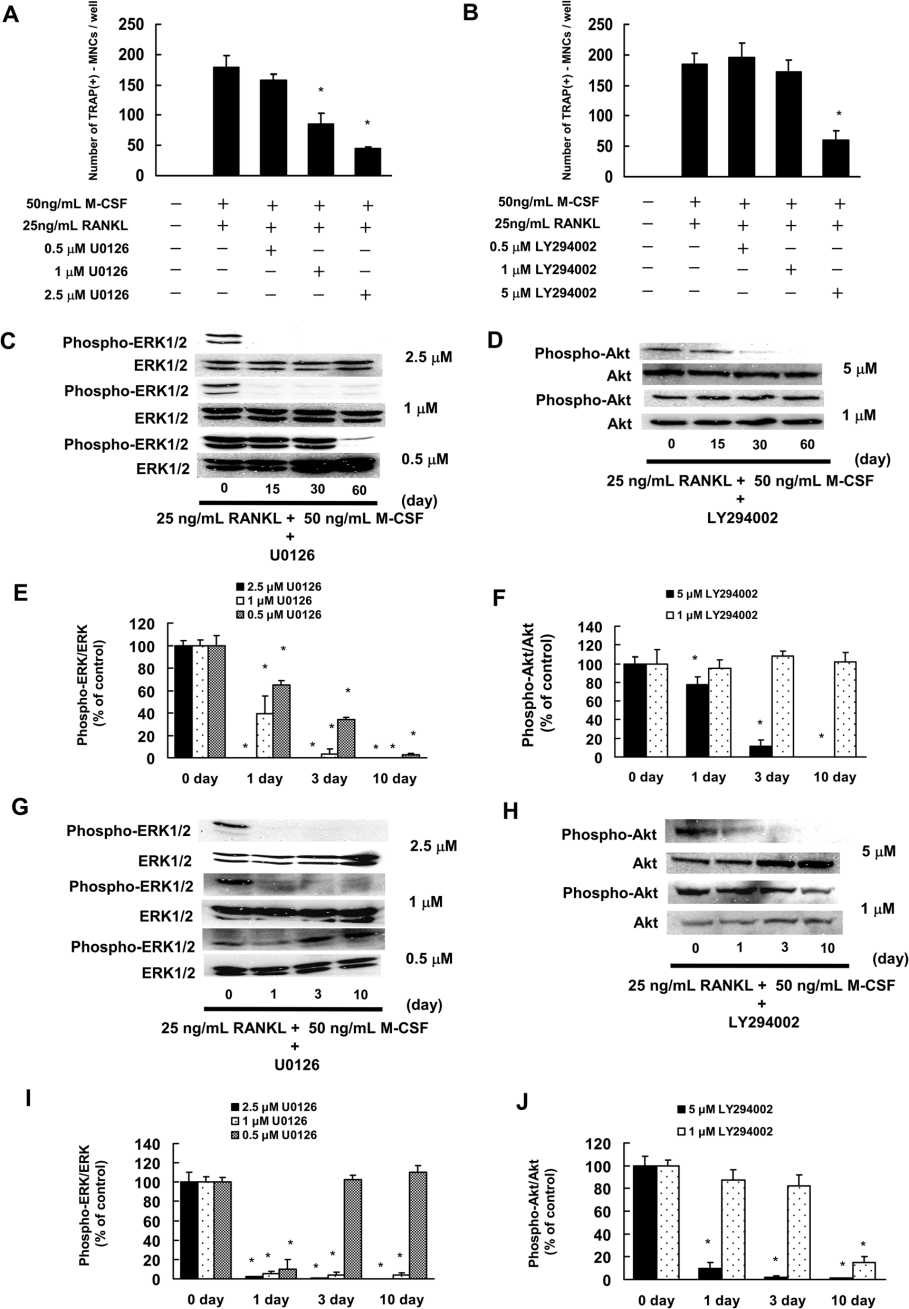
**U0126 (MEK1/2 inhibitor) or LY294002 (PI3K inhibitor) inhibited osteoclast formation. (A, B)** C7 cells were treated with 0.25, 0.5, 1, or 2.5 μM U0126 **(A)** or 0.5, 1, 2.5, or 5 μM LY294002 **(B)**. Cells receiving U0126 or LY294002 were cultured in the presence of 25 ng/mL RANKL plus 50 ng/mL M-CSF. Cultures were fed every 3 days by replacing with 500 μL of fresh medium, with or without U0126, LY294002, RANKL, and M-CSF. Cultures were fixed and stained for TRAP-positive multinucleated cells per well was counted. These results are representative of 5 independent experiments. *P < 0.01 compared to 25 ng/mL RANKL plus 50 ng/mL M-CSF administration. **(C, D)** C7 cells were treated with 0.5, 1, or 2.5 μM U0126 or 1 or 5 μM LY294002 **(D)** for 24 h. Cells were cultured in the presence of 25 ng/mL RANKL plus 50 ng/mL M-CSF for 15, 30, and 60 min. **(C)** C7 cells were treated with 0.5 μM minodronate for 24 h. Cells were cultured in the presence of 25 ng/mL RANKL plus 50 ng/mL M-CSF for 15, 30, and 60 min. **(E, F)** Quantification of the amount of phospho-ERK1/2 or phospho-Akt normalized to the amount of total ERK1/2 or Akt, respectively. The results are representative of 5 independent experiments. *P < 0.01 compared to controls. **(G, H)** C7 cells were treated with 0.5, 1, or 2.5 μM U0126 or 1 or 5 μM LY294002 **(D)** for 24 h. Cells were cultured in the presence of 25 ng/mL RANKL plus 50 ng/mL M-CSF for 1, 3, and 10 days. **(I, J)** Quantification of the amount of phospho-ERK1/2 or phospho-Akt normalized to the amount of total ERK1/2 or Akt, respectively. The results are representative of 5 independent experiments. *P < 0.01 compared to controls.

It is suggested that osteoclast formation is inhibited in C7 cells by prevention of GGPP biosynthesis in the mevalonate pathway, thereby blocking the signaling pathway through the MEK/ERK and PI3K/Akt pathways. Therefore, an attempt was made to confirm whether osteoclast formation was inhibited by inhibiting production of ERK1/2 and Akt. U0126, a MEK1/2 inhibitor, and LY294002, a PI3K inhibitor, were administered to C7 cells, and their effect on osteoclast formation was examined. U0126 and LY294002 inhibited osteoclast formation in a concentration-dependent manner (Figure 6A, B). Administration of U0126 showed a decrease in phosphorylated ERK1/2 15–60 min and 1–10 days after RANKL plus M-CSF treatments in a concentration-dependent manner. Administration of LY294002 showed a decrease in phosphorylated Akt 15–60 min and 1–10 days after RANKL plus M-CSF treatments in a concentration-dependent manner. U0126 (1 μM) or LY294002 (1 μM) appeared to decrease production of phosphorylated ERK1/2 or Akt to the same level as minodronate and alendronate (Figure 5C-F, Figure 5I-L, Figure 6C-F). These results suggested that 1 μM U0126 or 1 μM LY294002 inhibited the phosphorylation of ERK1/2 or Akt to the same degree as minodronate.

**Figure 7:**
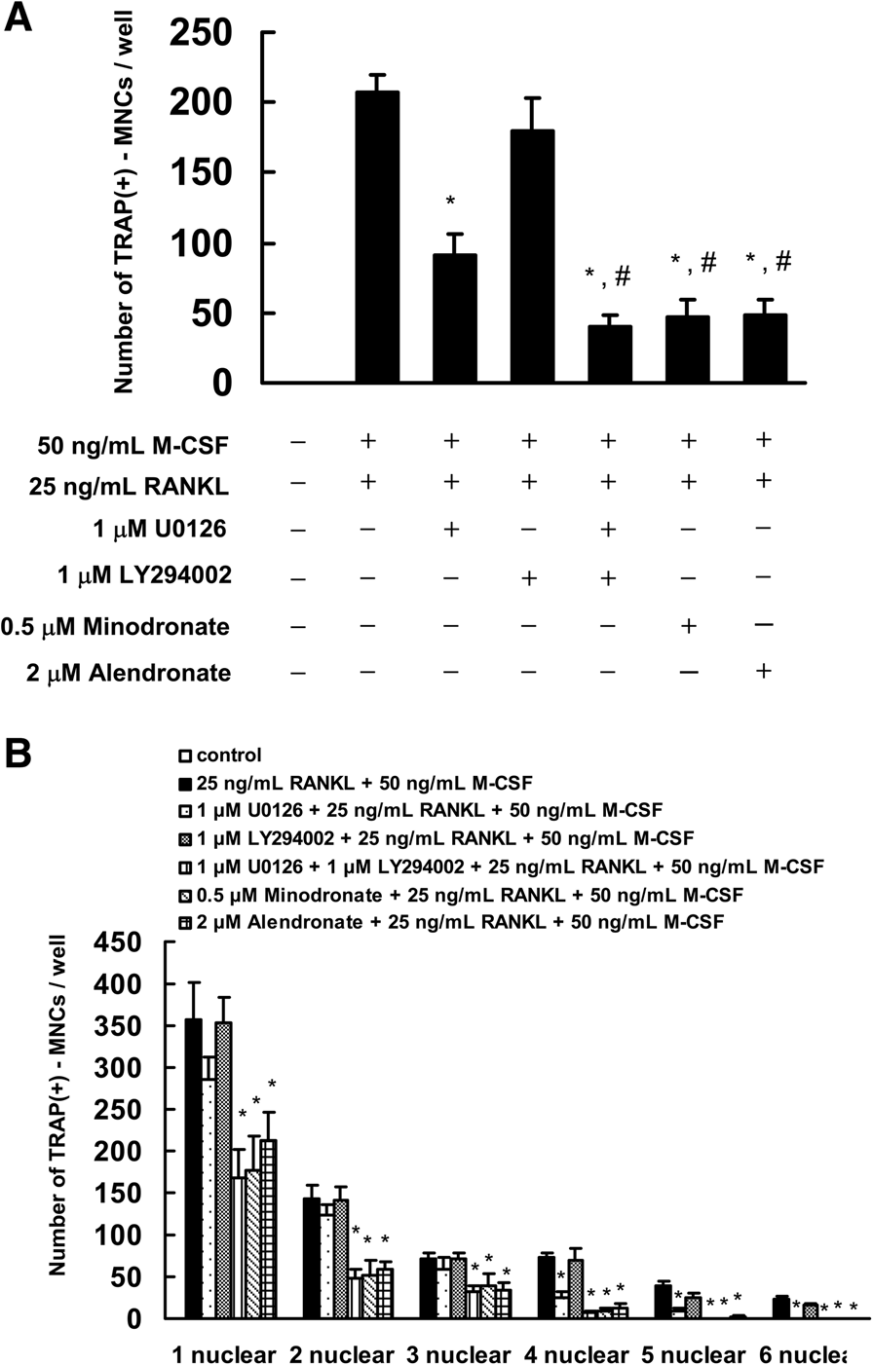
**Combined effect of U0126 and LY294002 inhibited the osteoclast formation. (A)** C7 cells were treated with 1 μM U0126 and 1 μM LY294002 for 24 hours. Cells were cultured in the presence of 25 ng/mL RANKL plus 50 ng/mL M-CSF. Cultures were fed every 3 days by replacing with 500 μL of fresh medium with or without minodronate, alendronate, U0126, LY294002, RANKL, and M-CSF. Cultures were fixed and stained for TRAP-positive multinucleated cells, and the number of cells per well was counted. *P < 0.01, compared to 25 ng/mL RANKL plus 50 ng/mL M-CSF administration. ^#^P < 0.01, compared to 1 μM U0126 administration. **(B)** Cultures were fixed and stained for TRAP-positive cells, and the number of cells per well was counted. These results are representative of 4 independent experiments. *P < 0.01 compared to 25 ng/mL RANKL plus 50 ng/mL M-CSF administration.

We next investigated whether a combination of 1 μM U0126 and 1 μM LY294002 inhibited osteoclast formation in C7 cells to the same level as minodronate. The combination of U0126 and LY294002 inhibited osteoclast formation and number of TRAP-positive fused cells compared with that of administration of U0126 or LY294002 alone. Moreover, the combinatorial effect of 1 μM U0126 and 1 μM LY294002 on osteoclast formation and number of TRAP-positive fused cells declined to the same level as minodronate and alendronate (Figure 7). These results suggested that minodronate and alendronate inhibited osteoclast formation through the suppression of ERK1/2 and Akt activation.

## Discussion

In this study, we demonstrated that minodronate and alendronate inhibit osteoclast formation through the suppression of cell fusion induced by RANKL plus M-CSF or RANKL in cultures of osteoclast precursor cells, indicating that the mechanism of action is due to the inhibition of GGPP biosynthesis. Coxon et al. reported that a geranylgeranyl transferase inhibitor, but not a farnesyl transferase inhibitor, inhibits the differentiation, function, and survival of multinucleated osteoclasts in bone marrow cultures [26]. These findings suggest that the geranylgeranyl small GTPs are important for the osteoclast formation.

We observed that ERK1/2 and Akt were activated by the treatment of RANKL plus M-CSF, whereas the activation was inhibited by the administration of minodronate and alendronate. We also observed that U0126, a MEK1/2 inhibitor, and LY294002, a PI3K inhibitor, inhibited osteoclast formation in a concentration-dependent manner. Furthermore, combined administration of 1 μM U0126 and 1 μM LY294002 inhibited osteoclast formation in C7 cells, as observed with minodronate and alendronate. These results suggested that the MEK/ERK and PI3K/Akt pathways are required for osteoclast formation, which is also supported by the above-mentioned RANKL plus M-CSF actions. These findings demonstrated that minodronate and alendronate could inhibit osteoclast formation.

It was reported that RANK signaling activated transcriptional factors through several signal transduction pathways [27]. Among these factors, the activation of those closely linked to osteoclast formation is regulated by the MEK/ERK pathway. Downstream targets of ERK1/2 induce and activate c-Fos, a transcription factor for AP-1, which is essential for osteoclastogenesis [27]. Previous studies indicated that M-CSF stimulated ERK1/2 activation in osteoclast precursor cells [28]. These reported findings raise the possibility that ERK1/2 activation is necessary for osteoclast formation. Moreover, we demonstrated that minodronate and alendronate inhibit osteoclast formation through suppression of ERK1/2 activation. These results suggest that minodronate and alendronate are not only potentially useful as an anti-resorptive agent for inhibiting osteoclast formation, but also for inducing apoptosis in osteoclasts.

In the present study, we found that minodronate and alendronate at lower concentrations of 0.25 and 1 μM or 1 and 5 μM inhibited osteoclast formation via suppression of cell fusion in C7 or RAW264.7 cells for 12 days or 7 days, respectively. It has been reported that zolendronate at a concentration of 30 μM for 3 days inhibited the differentiation of osteoclast and osteoclast precursor cell migration in RAW264.7 cells and in mouse bone marrow cells [29]. In addition, various N-BPs were shown to inhibit osteoclast differentiation at a lower concentration of 0.1 μM in RAW264.7 cells for 6 days [30]. The inhibitory effect of N-BPs on osteoclast formation at different concentrations may be due to treatment for different amounts of time in osteoclast precursor cells. Collectively, these findings suggest that N-BPs suppress osteoclast formation via inhibition of cell fusion and migration.

Myeloma, breast, and lung cancer show osteolytic changes in bone [31,32]. Osteolysis is an essential component of the progression of every primary or metastatic tumor in bone [31]. BPs have been reported to have direct antitumor effects [14,18,22,23,25]. The antitumor activities of BPs include induction of tumor apoptosis, inhibition of tumor cell proliferation, decreased tumor cell adhesion, and invasion into bone [33,34]. Minodronate has reported to inhibit tumor cell metastasis in bone and to suppress the metastasis-related bone destruction and osteoclast activity [35-38]. In addition, alendronate reduced ongoing and movement-evoked bone cancer pain, bone destruction, and the destruction of sensory nerve fibers that innervate the bone [39]. Moreover, alendronate reduced the formation of bone metastasis, and in addition to paclitaxel, prevented the formation of bone metastasis, non-osseous metastasis, and increased survival in prostate cancer murine models [40]. These findings suggest that minodronate and alendronate may suppress cancer-related bone destruction via inhibition of osteoclast formation, osteoclast activity, and tumor cell metastasis.

## Conclusion

In conclusion, we observed the inhibitory action of minodronate and alendronate on osteoclast formation through suppression of ERK1/2 and Akt activations in a macrophage-like cell line of C7 cells. This finding may indicate the potential efficacy of minodronate and alendronate in the therapy of metabolic bone diseases.

## Abbreviations

GGPP: Geranylgeranyl pyrophosphate; ERK: Extracellular signal-regulated kinase; MEK: Mitogen protein kinase; PI3K: Phosphatidylinositol 3-kinase; M-CSF: Macrophage-colony stimulating factor; RANKL: Receptor activator of NF-κB ligand; MAPK: Mitogen-activated protein kinase; N-BPs: Nitrogen-containing bisphosphonate; PBS: Phosphate-buffered saline; FPP: Farnesyl pyrophosphate; FOH: Farnesol; GGOH: Geranylgeraniol; TRAP: Tartrate-resistant acid phosphatase.

## Competing interests

The authors declare that they have no competing interests.

## Authors’ contributions

MT carried out analysis of TRAP staining, cell viability assay, western blotting analysis, statistical analysis, and drafted the manuscript. MK carried out analysis of cell viability assay and western blotting analysis. TI, TS, MI, KS, and HS carried out western blotting analysis. TT, NO, KM, and DF carried out analysis of TRAP staining. JM, KS, and TS contributed to statistical analyses. SN designed the experiments and revised the manuscript. All authors read and approved the final manuscript.

## References

[B1] Roodman GD Advances in bone biology: the osteoclast Endocr Rev 1996 17 308 332 8854048 10.1210/edrv-17-4-308

[B2] Suda T Takahashi N Udagawa N Jimi E Gillespie MT Martin TJ Modulation of osteoclast differentiation and function by the new members of the tumor necrosis factor receptor and ligand families Endocr Rev 1999 20 345 357 10.1210/edrv.20.3.0367 10368775

[B3] Teitelbaum SL Bone resorption by osteoclasts Science 2000 289 1504 1508 10.1126/science.289.5484.1504 10968780

[B4] Karsenty G Wagner EF Reaching a genetic and molecular understanding of skeletal development Dev Cell 2002 2 389 406 10.1016/S1534-5807(02)00157-0 11970890

[B5] Boyle WJ Simonet WS Lacey DL Osteoclast differentiation and activation Nature 2003 423 337 342 10.1038/nature01658 12748652

[B6] Teitelbaum SL Ross FP Genetic regulation of osteoclast development and function Nat Rev Genet 2003 4 638 649 10.1038/nrg1122 12897775

[B7] Lerner UH New molecules in the tumor necrosis factor ligand and receptor superfamilies with importance for physiological and pathological bone resorption Crit Rev Oral Biol Med 2004 15 64 81 10.1177/154411130401500202 15059943

[B8] Yoshida H Hayashi S Kunisada T Ogawa M Nishikawa S Okamura H Sudo T Shultz LD Nishikawa S The murine mutation osteopetrosis is in the coding region of the macrophage colony stimulating factor gene Nature 1990 345 442 443 10.1038/345442a0 2188141

[B9] Kong YY Yoshida H Sarosi I Tan HL Timms E Capparelli C Morony S Oliveira-dos-Santos AJ Van G Itie A Khoo W Wakeham A Dunstan CR Lacey DL Mak TW Boyle WJ Penninger JM OPGL is a key regulator of osteoclastogenesis, lymphocyte development and lymph-node organogenesis Nature 1999 397 315 323 10.1038/16852 9950424

[B10] Takayanagi H Mechanistic insight into osteoclast differentiation in osteoimmunology J Mol Med 2005 83 170 179 10.1007/s00109-004-0612-6 15776286

[B11] Dai XM Ryan GR Hapel AJ Dominguez MG Russell RG Kapp S Sylvestre V Stanley ER Targeted disruption of the mouse colony-stimulating factor 1 receptor gene results in osteopetrosis, mononuclear phagocyte deficiency, increased primitive progenitor cell frequencies, and reproductive defects Blood 2002 99 111 120 10.1182/blood.V99.1.111 11756160

[B12] Rogers MJ Gordon S Benford HL Coxon FP Luckman SP Monkkonen J Frith JC Cellular and molecular mechanisms of action of bisphosphonates Cancer 2000 88 2961 2978 10.1002/1097-0142(20000615)88:12+<2961::AID-CNCR12>3.0.CO;2-L 10898340

[B13] Keller RK Fliesler SJ Mechanism of aminobisphosphonate action: characterization of alendronate inhibition of the isoprenoid pathway Biochem Biophys Res Commun 1999 266 560 563 10.1006/bbrc.1999.1849 10600541

[B14] Nishida S Fujii Y Yoshioka S Kikuichi S Tsubaki M Irimajiri K A new bisphosphonate, YM529 induces apoptosis in HL60 cells by decreasing phosphorylation of single survival signal ERK Life Sci 2003 73 2655 2664 10.1016/S0024-3205(03)00664-7 13679234

[B15] Tsubaki M Kato C Nishinobo M Ogaki M Satou T Ito T Kusunoki T Fujiwara K Yamazoe Y Nishida S Nitrogen-containing bisphosphonate, YM529/ONO-5920, inhibits macrophage inflammatory protein 1 alpha expression and secretion in mouse myeloma cells Cancer Sci 2008 99 152 158 17979996 10.1111/j.1349-7006.2007.00651.x PMC11159071

[B16] Nishida S Tsubaki M Hoshino M Namimatsu A Uji H Yoshioka S Tanimori Y Yanae M Iwaki M Irimajiri K Nitrogen-containing bisphosphonate, YM529/ONO-5920 (a novel minodronic acid), inhibits RANKL expression in a cultured bone marrow stromal cell line ST2 Biochem Biophys Res Commun 2005 328 91 97 10.1016/j.bbrc.2004.12.145 15670755

[B17] Fisher JE Rogers MJ Halasy JM Luckman SP Hughes DE Masarachia PJ Wesolowski G Russell RG Rodan GA Reszka AA Alendronate mechanism of action: geranylgeraniol, an intermediate in the mevalonate pathway, prevents inhibition of osteoclast formation, bone resorption, and kinase activation in vitro Proc Natl Acad Sci USA 1999 96 133 138 10.1073/pnas.96.1.133 9874784 PMC15105

[B18] Rogers MJ Crockett JC Coxon FP Mönkkönen J Biochemical and molecular mechanisms of action of bisphosphonates Bone 2011 49 34 41 10.1016/j.bone.2010.11.008 21111853

[B19] Miyamoto A Kunisada T Hemmi H Yamane T Yasuda H Miyake K Yamazaki H Hayashi SI Establishment and characterization of an immortal macrophage-like cell line inducible to differentiate to osteoclasts Biochem Biophys Res Commun 1998 242 703 709 10.1006/bbrc.1997.8046 9464281

[B20] Tsubaki M Kato C Isono A Kaneko J Isozaki M Satou T Itoh T Kidera Y Tanimori Y Yanae M Nishida S Macrophage inflammatory protein-1α induces osteoclast formation by activation of the MEK/ERK/c-Fos pathway and inhibition of the p38MAPK/IRF-3/IFN-β pathway J Cell Biochem 2010 111 1661 1672 10.1002/jcb.22907 21053363

[B21] Russell RG Rogers MJ Bisphosphonates: from the laboratory to the clinic and back again Bone 1999 25 97 106 10.1016/S8756-3282(99)00116-7 10423031

[B22] Tanimori Y Tsubaki M Yamazoe Y Satou T Itoh T Kidera Y Yanae M Yamamoto C Kaneko J Nishida S Nitrogen-containing bisphosphonate, YM529/ONO-5920, inhibits tumor metastasis in mouse melanoma through suppression of the Rho/ROCK pathway Clin Exp Metastasis 2010 27 529 538 10.1007/s10585-010-9342-z 20632074

[B23] Tsubaki M Satou T Itoh T Imano M Ogaki M Yanae M Nishida S Reduction of metastasis, cell invasion, and adhesion in mouse osteosarcoma by YM529/ONO-5920-induced blockade of the Ras/MEK/ERK and Ras/PI3K/Akt pathway Toxicol Appl Pharmacol 2012 259 402 410 10.1016/j.taap.2012.01.024 22326785

[B24] Tsubaki M Satou T Itoh T Imano M Yanae M Kato C Takagoshi R Komai M Nishida S Bisphosphonate- and statin-induced enhancement of OPG expression and inhibition of CD9, M-CSF, and RANKL expressions via inhibition of the Ras/MEK/ERK pathway and activation of p38MAPK in mouse bone marrow stromal cell line ST2 Mol Cell Endocrinol 2012 361 219 231 10.1016/j.mce.2012.05.002 22579611

[B25] Tsubaki M Itoh T Satou T Imano M Komai M Ogawa N Mukai J Nishida S Nitrogen-containing bisphosphonates induce apoptosis of hematopoietic tumor cells via inhibition of Ras signaling pathways and Bim-mediated activation of the intrinsic apoptotic pathway Biochem Pharmacol 2013 85 163 172 10.1016/j.bcp.2012.10.009 23085435

[B26] Coxon FP Helfrich MH Van’t Hof R Sebti S Ralston SH Hamilton A Rogers MJ Protein geranylgeranylation is required for osteoclast formation, function, and survival: inhibition by bisphosphonates and GGTI-298 J Bone Miner Res 2000 15 1467 1476 10.1359/jbmr.2000.15.8.1467 10934645

[B27] Lee ZH Kim HH Signal transduction by receptor activator of nuclear factor kappa B in osteoclasts Biochem Biophys Res Commun 2003 305 211 214 10.1016/S0006-291X(03)00695-8 12745060

[B28] Faccio R Takeshita S Zallone A Ross FP Teitelbaum SL c-Fms and the alphavbeta3 integrin collaborate during osteoclast differentiation J Clin Invest 2003 111 749 758 10.1172/JCI200316924 12618529 PMC151897

[B29] Kimachi K Kajiya H Nakayama S Ikebe T Okabe K Zoledronic acid inhibits RANK expression and migration of osteoclast precursors during osteoclastogenesis Naunyn Schmiedebergs Arch Pharmacol 2011 383 297 308 10.1007/s00210-010-0596-4 21225243

[B30] Abe K Yoshimura Y Deyama Y Kikuiri T Hasegawa T Tei K Shinoda H Suzuki K Kitagawa Y Effects of bisphosphonates on osteoclastogenesis in RAW264.7 cells Int J Mol Med 2012 29 1007 1015 22447156 10.3892/ijmm.2012.952

[B31] Clezardin P Teti A Bone metastasis: pathogenesis and therapeutic implications Clin Exp Metastasis 2007 24 599 608 10.1007/s10585-007-9112-8 18008175

[B32] Esteve FR Roodman GD Pathophysiology of myeloma bone disease Best Pract Res Clin Haematol 2007 20 613 624 10.1016/j.beha.2007.08.003 18070709

[B33] Boissier S Ferreras M Peyruchaud O Magnetto S Ebetino FH Colombel M Delmas P Delaissé JM Clézardin P Bisphosphonates inhibit breast and prostate carcinoma cell invasion, an early event in the formation of bone metastases Cancer Res 2000 60 2949 2954 10850442

[B34] Clézardin P Ebetino FH Fournier PG Bisphosphonates and cancer-induced bone disease: beyond their antiresorptive activity Cancer Res 2005 65 4971 4974 10.1158/0008-5472.CAN-05-0264 15958534

[B35] Miwa S Mizokami A Keller ET Taichman R Zhang J Namiki M The bisphosphonate YM529 inhibits osteolytic and osteoblastic changes and CXCR-4-induced invasion in prostate cancer Cancer Res 2005 65 8818 8825 10.1158/0008-5472.CAN-05-0540 16204052

[B36] Cui N Nomura T Noma H Yokoo K Takagi R Hashimoto S Okamoto M Sato M Yu G Guo C Shibahala T Effect of YM529 on a model of mandibular invasion by oral squamous cell carcinoma in mice Clin Cancer Res 2005 11 2713 2719 10.1158/1078-0432.CCR-04-1767 15814653

[B37] Yonou H Ochiai A Ashimine S Maeda H Horiguchi Y Yoshioka K Ogawa Y Hatano T Tachibana M The bisphosphonate YM529 inhibits osteoblastic bone tumor proliferation of prostate cancer Prostate 2007 67 999 1009 10.1002/pros.20592 17440967

[B38] Zhang H Yano S Miki T Goto H Kanematsu T Muguruma H Uehara H Sone S A novel bisphosphonate minodronate (YM529) specifically inhibits osteolytic bone metastasis produced by human small-cell lung cancer cells in NK-cell depleted SCID mice Clin Exp Metastasis 2003 20 153 159 10.1023/A:1022621622063 12705636

[B39] Sevcik MA Luger NM Mach DB Sabino MA Peters CM Ghilardi JR Schwei MJ Röhrich H De Felipe C Kuskowski MA Mantyh PW Bone cancer pain: the effects of the bisphosphonate alendronate on pain, skeletal remodeling, tumor growth and tumor necrosis Pain 2004 111 169 180 10.1016/j.pain.2004.06.015 15327821

[B40] Neville-Webbe HL Coleman RE Bisphosphonates and RANK ligand inhibitors for the treatment and prevention of metastatic bone disease Eur J Cancer 2010 46 1211 1222 10.1016/j.ejca.2010.02.041 20347292

